# Data-Driven Inference Reveals Distinct and Conserved Dynamic Pathways of Tool Use Emergence across Animal Taxa

**DOI:** 10.1016/j.isci.2020.101245

**Published:** 2020-06-09

**Authors:** Iain G. Johnston, Ellen C. Røyrvik

**Affiliations:** 1 Department of Mathematics, Faculty of Mathematics and Natural Sciences, University of Bergen, Bergen, Norway; 2 Alan Turing Institute, London, UK; 3 Department of Clinical Sciences, University of Bergen, Bergen, Norway

**Keywords:** Biocomputational Method, Bioinformatics, Ethology, Evolutionary Biology

## Abstract

Tool use is a striking aspect of animal behavior, but it is hard to infer how the capacity for different types of tool use emerged across animal taxa. Here we address this question with HyperTraPS, a statistical approach that uses contemporary observations to infer the likely orderings in which different types of tool use (digging, reaching, and more) were historically acquired. Strikingly, despite differences linked to environment and family, many similarities in these appear across animal taxa, suggesting some universality in the process of tool use acquisition across different animals and environments. Four broad classes of tool use are supported, progressing from simple object manipulations (acquired relatively early) to more complex interactions and abstractions (acquired relatively late or not at all). This data-driven, comparative approach supports existing and suggests new mechanistic hypotheses, predicts future and possible unobserved behaviors, and sheds light on patterns of tool use emergence across animals.

## Highlights

• Historical pathways of tool use acquisition inferred from large catalog of data
• Striking similarities in acquisition pathways across environments and lineages
• Acquisitions of different modes of tool use broadly follow conceptual complexity
• Wild/domestic differences and predictions of future/unobserved behaviors quantified

## Graphical Abstract

**Figure:**
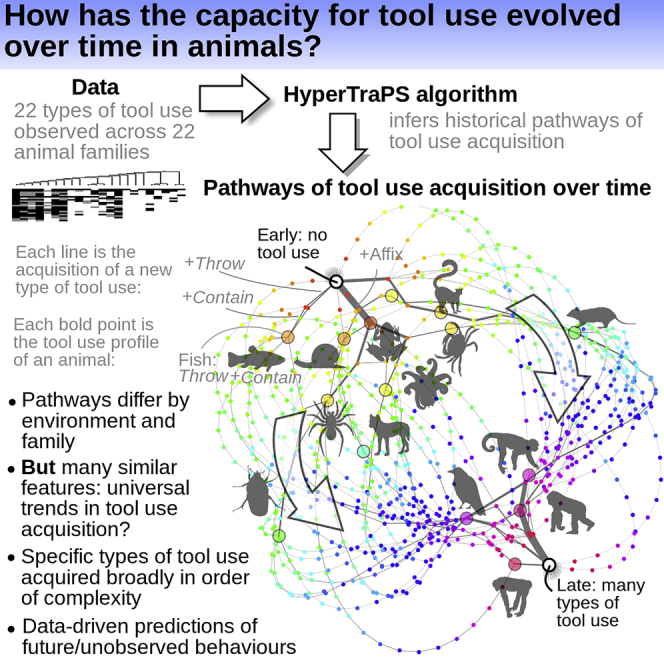

## Introduction

Tool use forms a central part of the behavioral repertoire of many phylogenetically diverse animals. The study of tool use can reveal principles of behavioral ontogeny, ecology, and cognition (Tomasello and Call, 1997, Povinelli, 2000, Emery and Clayton, 2009, Seed and Byrne, 2010), as well as the evolution of material culture (Van Schaik et al., 1999) and indeed human behavior (Byrne, 2004). The complicated processes involved in different types of tool use also place it as a valuable but underexplored case study in the evolution of biological complexity (Heylighen et al., 1999, McShea, 1991).

Despite this interdisciplinary centrality, the observation and quantitative analysis of the emergence of tool use present challenges. Here and throughout, we use *emergence* to refer to the appearance over time of the capacity for particular modes of tool use in members of a given animal family. Although detailed catalogs of observed tool use behavior in animals exist and continue to expand (Beck, 1980, Shumaker et al., 2011), these observations typically constitute records of tool use instances in individual species, lacking a unifying analytical framework. Although some taxa, notably primates and birds (Emery and Clayton, 2009, Shumaker et al., 2011), have been studied in particular depth (Seed and Byrne, 2010), data-driven approaches amalgamating tool use information across a broad range of animal taxa are more limited. Among these comparative data approaches, Van Schaik et al. (1999) consider a cross-section of observations across primate species to propose a qualitative model for the features required for development of tool use. This model consists of a hierarchical set of behavioral, social, and intelligence traits, which were explored through experiment and assigned a putative phylogenetic embedding. Another comparative analysis (Hunt et al., 2013) estimated the number of independent occurrences/innovative events of tool use in different taxa, scaled by the number of species in a given taxon. Not unexpectedly, primates have the highest degree of independent occurrences, an order of magnitude higher than the next most innovative taxon, birds. Comparisons across two closely related primate species (bonobos and chimpanzees) have also been used to explore possible evolutionary inheritance of tool use (Gruber et al., 2010). A review of tool use across birds and mammals (Emery and Clayton, 2009) considered the relationship between tool use and physical intelligence, finding that despite a correlation between tool use and brain size in birds and primates (Lefebvre et al., 2002, Reader and Laland, 2002), tool use is not a clear predictor of physical intelligence. In agreement with this picture, Hunt et al. postulate a more important role for working memory in propensity for tool use (Hunt et al., 2013).

The complexity and nuance of tool use classification (Beck, 1980, Hunt et al., 2013, Shumaker et al., 2011, St Amant and Horton, 2008) and the numerous types of behavior observed in different lineages can challenge the expansion of such comparative approaches. Specifically, it remains unclear how best to harness and unify the available “snapshots” of specific tool use instances in diverse species to provide an understanding of the emergence of tool use across animals. The large and debated number of different modes of observed tool use (for example, *dig*, *scratch*, *reach*, and more [Table S1]), and their potential interactions, present a substantial hurdle to building an overall theoretical picture of this emergence.

In a groundbreaking early catalog of observations, Beck (1980) notes that “the evolution of tool behavior appears to represent a complex *parallelism and convergence* that is resistant to a simple phyletic analysis” [emphasis added]. To quantitatively resolve such evolutionary questions regarding the parallel acquisition of many discrete, coupled traits, the HyperTraPS (hypercubic transition path sampling) algorithm has recently been developed (Johnston and Williams, 2016, Greenbury et al., 2019, Williams et al., 2013).

In this context, HyperTraPS takes a set of observations of features across organisms and infers the possible orderings in which these features were most likely acquired. For example, if many species employ *affix* but few employ *symbolize*, we may infer that the capacity to *affix* is likely to be acquired before the capacity to *symbolize*. Of course, evolutionary history influences the details of this inference, and is accounted for in HyperTraPS (Johnston and Williams, 2016, Greenbury et al., 2019). More generally, HyperTraPS efficiently infers the historical orderings by which a set of features may be progressively acquired in a system—in this study, the orderings in which the capacities for different modes of tool use may be acquired over evolutionary time, which we refer to as *dynamic pathways*. HyperTraPS can infer dynamics of, and influences between, dozens of features that may or may not interact, and, importantly, can harness diverse contemporary observations (for example, patterns of tool use modes in different animal families) to infer past dynamic pathways with quantified uncertainty.

Here, we use a detailed compilation of observed tool use behavior across the animal kingdom (Shumaker et al., 2011) in conjunction with HyperTraPS to reveal the structure of, and variability in, dynamic pathways of animal tool use emergence. We identify several distinct and well-defined pathways of tool use emergence, conserved across animal taxa, and characterize their links with environmental and taxonomic features. We find that the dynamic acquisition of tool use modes is qualitatively linked with the complexity of interactions that they involve, and use this Bayesian framework to make predictions about future and unobserved modes of tool use behavior.

## Results

### Dynamic Pathways of Tool Use Emergence

**Figure 1:**
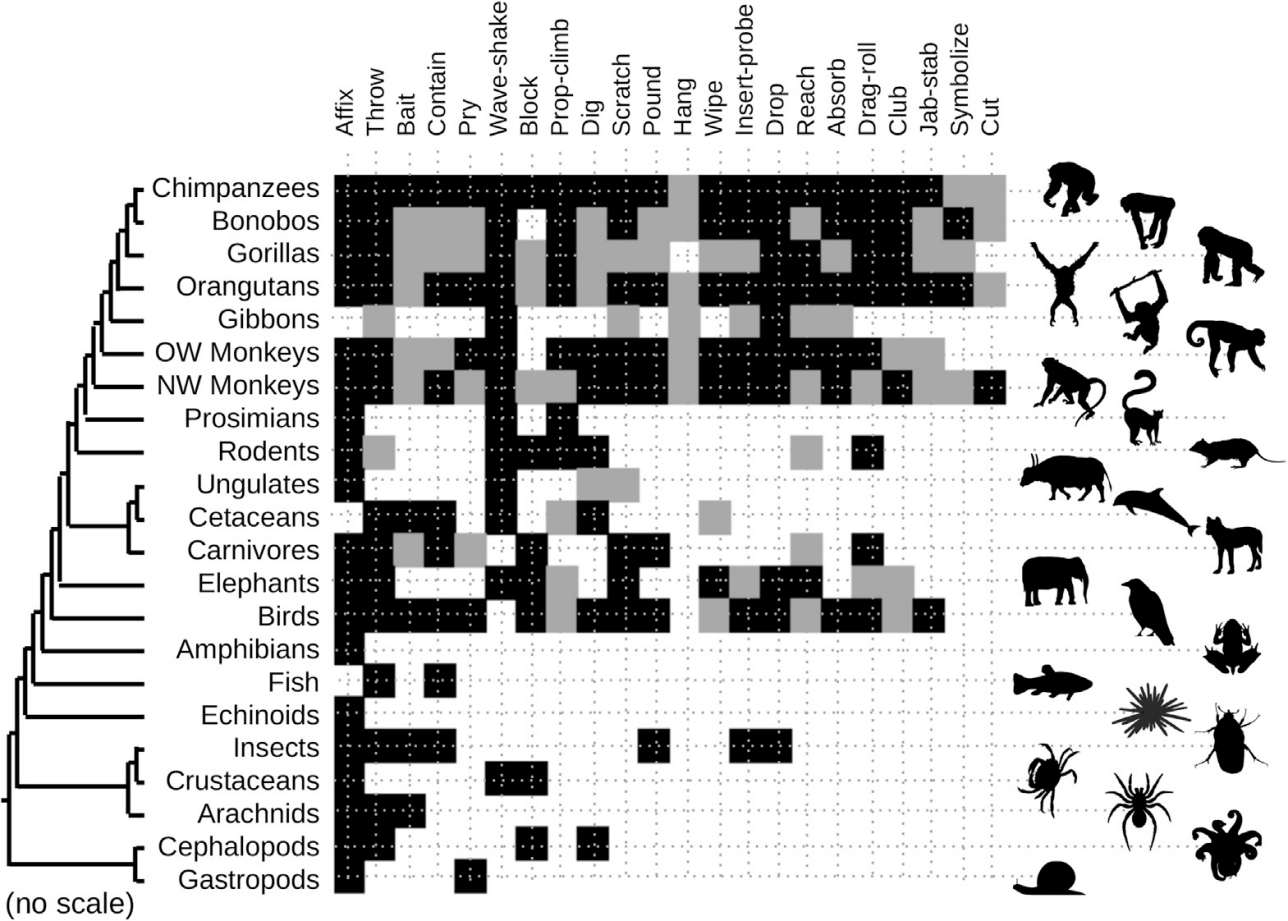
Observations of Tool Use Modes across Taxa Horizontal axis gives the 22 modes of tool use classified in Shumaker et al. (2011). Vertical axis gives groups in which these modes have been observed, connected by a coarse-grained taxonomy from NCBI Taxonomy Common Tree (Federhen, 2011). After Shumaker et al. (2011), a black pixel denotes an observation of a mode of tool use in the wild by a taxonomic group; a gray pixel denotes an observation in which human influence cannot be discounted; a white pixel denotes absence of that observation. NW, New World; OW, Old World.

We obtained data on observed modes of tool use across animal taxa from Shumaker et al. (2011) and used the NCBI Taxonomy Common Tree tool (Federhen, 2011) to embed these on a putative phylogeny (Figure 1). We note that these modes of tool use are open to interpretation; we choose the modes from Shumaker et al. (2011) as a systematically constructed set that encompasses a very broad range of possible tool use behaviors, and give that study's specific definitions of these modes and associated terms in Table S1. Tool use varies across individuals and populations within species according to ecological and other features (Shumaker et al., 2011) (for example, Fujii et al., 2015, Humle and Matsuzawa, 2001, McGrew, 1992, Ottoni and Izar, 2008), so an observation of a particular mode of tool use in a particular species demonstrates that that species has the capacity to employ that mode of tool use but does not imply that all individuals within that species will employ it. We take this coarse-grained perspective to allow a comparative analysis across different animal species, noting that our results can be interpreted with substantial flexibility in the subset of these modes in which the reader is interested (see below and Discussion). The structure of these data immediately highlights the diverse modes of tool use observed in primates and (presenting more of a taxonomic outlier) birds (Van Schaik et al., 1999, Emery and Clayton, 2009).

We used HyperTraPS to infer the dynamic pathways by which tool use has emerged across animal taxa. HyperTraPS considers different “states” corresponding to every possible pattern of tool use (that is, every combination of presence or absence for each mode). Each state is labeled by L=22 binary digits denoting whether a particular mode of tool use is associated with that state or not. For example, the state *1000 …* corresponds to only the first mode of tool use (*affix* in the default ordering, shown in Figure 1) being observed in a given species. Modes of tool use are modeled as irreversibly acquired once “discovered.” HyperTraPS functions by constructing a model of how each state (each pattern of acquired tool use modes) may transition to any new state over evolutionary time and using observations to parameterize this model, so that the most likely transitions between states are supported. HyperTraPS thus learns the probabilities of possible evolutionary transitions by which an ancestor demonstrating no modes of tool use (state *0000 …*) can acquire the various modes that lead to patterns observed in biology. This approach is Bayesian, with the output consisting of posterior probability distributions over the transition-rate parameters in this model.

These output distributions can be interpreted in a number of ways. First, as a set of specific pathways (for example, first *affix*, then *throw*, then *pry*, and so on), each with an associated probability of being the true order in which a given evolutionary lineage acquired modes of tool use. Second, as a summary of “acquisition propensities” over these pathways, reporting the probability that a given mode is acquired at a given ordering in an evolutionary lineage. Here, a given mode may display largely early acquisition (a high probability that it is acquired before other modes in any given evolutionary lineage), late acquisition (a high probability that it is acquired after other modes, if at all), or other more complex distributions of acquisition ordering.

**Figure 2:**
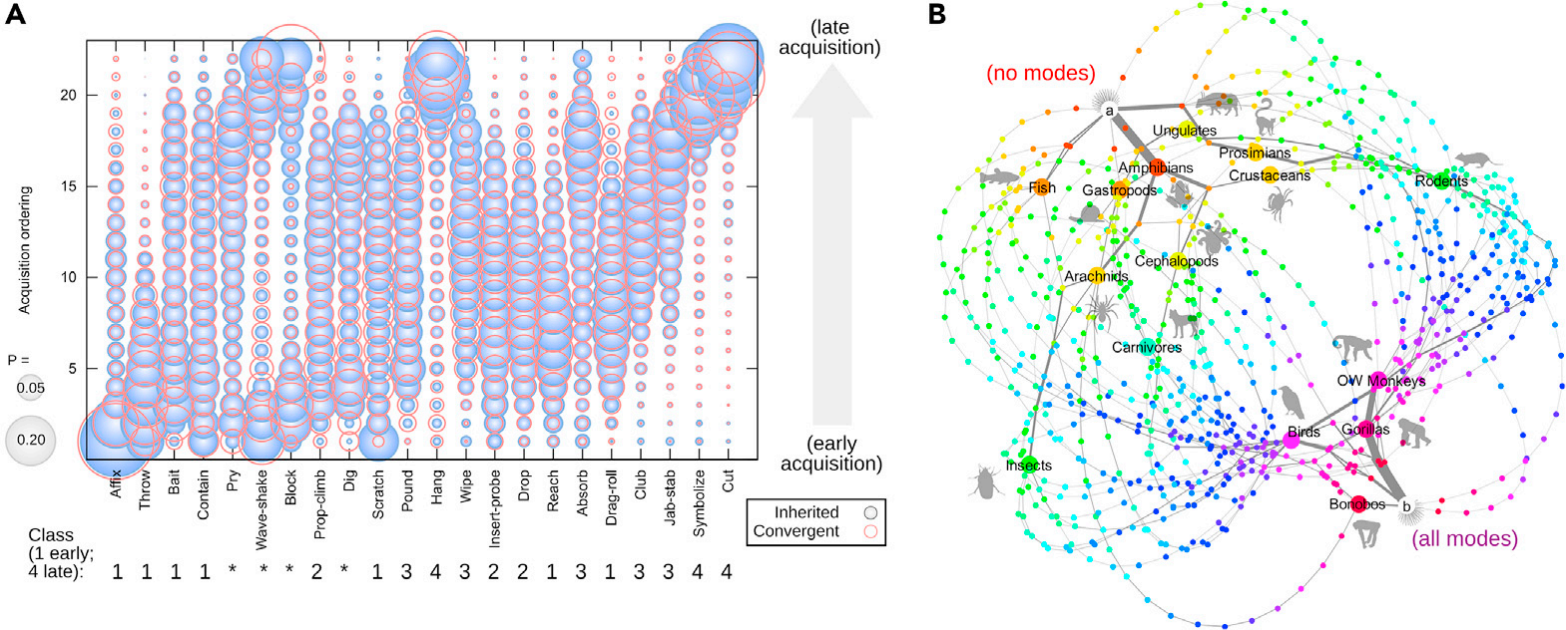
Inferred Pathways of Tool Use Emergence across Taxa (A) Posterior orderings of mode acquisition. The radius of a disk is proportional to the posterior probability that a given mode (horizontal axis) is acquired at a given step (vertical axis) in a pathway starting with no acquired modes. Blue disks give posteriors from inference given the phylogenetic relationship in Figure 1; red circles give (similar) posteriors from inference assuming convergent acquisitions (independent taxa). Numbers correspond to “classes” in the text, distinguished by higher probabilities of early (1) to late (4) acquisition; asterisks denote notably bimodal distributions. (B) High-probability subset of pathways through the hypercubic space of tool mode acquisitions. Each node is a state corresponding to a specific pattern of mode presence or absences. State *a* is the state of no modes; state *b* is the state of all modes acquired (thus including chimpanzees, orangutans, NW monkeys). Nodes corresponding to different observed taxa are highlighted; nodes are colored red-violet according to number of acquired modes. Edge width is proportional to the posterior probability of the corresponding transition between states. Two pathway groups closely following state *a* can be observed, which further branch into pathways occupying the core and periphery of the plot. NW, New World; OW, Old World.

Figure 2 shows the inferred transition probabilities from our tool use dataset in these two ways. Figure 2A gives the probabilities with which each given mode of tool use is the *n*th mode to be acquired, over the possible pathways learned from the data. Clear structure is visible, with some modes having a high probability of early acquisition, some intermediate, and some late; we will discuss this structure in more detail in the next section. Figure 2B illustrates specific high-probability pathways through the (hypercubic) space connecting all possible states. This embedding clearly shows a branched structure in emergence pathways. Several competing branches (occupied by, for example, amphibians and gastropods, or ungulates and prosimians) diverge at early stages of tool use acquisition and further disperse into more specific pathways occupied by other groups (for example, insects and arachnids, or rodents). These further pathways broadly divide the hypercubic space into two regions: one (visible around the top right in Figure 2B) more enriched for mammalian observations and the other (around the bottom left in Figure 2B) enriched for non-mammalian groups. Some fanning out of individual pathways occurs in regions of the hypercube between two observations: this fanning reflects the fact that several possible pathways are comparably likely between these observations.

These data must be viewed with several potentially confounding factors in mind. Beck (1980) draws explicit attention to the fact that the behavior of every taxon has not been studied with equal effort, by observers with equal sensitivity to tool behavior, or under conditions equally favorable for observing tool behavior (although the importance of these sampling effects has been questioned, particularly in avian research [Lefebvre and Nicolakakis, 2000]). Human influence may also itself affect the dynamics of tool use emergence. Furthermore, the phylogenetic embedding of these observations raises a degree of freedom: should the same observation in sibling clades be treated as evidence of convergent evolution or an inherited trait? And how can we incorporate the markedly different taxonomic levels at which observations are reported into our analysis?

We first address the question of inherited versus convergent evolution. Rather than attempting to find the specific most supported distribution of inherited and convergent traits, we instead consider the two limiting cases: first, where all traits shared across sibling clades are assumed to be inherited, and second, where all traits in each observed clade are assumed to have been acquired independently and convergently. As seen in Figure 2A, the inferred dynamics of trait acquisition do not display dramatic differences in these two limiting cases; their general properties can thus be assumed to hold regardless of the true inheritance patterns. The major effect distinguishing the inherited and convergent pictures is associated with primate-orientated modes, particularly *block* and *hang*. As the convergent picture treats each primate clade independently, primate-specific observations are weighted more heavily in this picture, leading to the observed (minor) reweighting of the posteriors.

To account for the fact that not all modes of behavior may have been observed by humans in all species, we next asked how robust our observations were to missing and erroneous observations. To this end, we produced several artificially resampled datasets. First, random additional positives were inserted into the observed data. Second, random perturbations were made to the observation set. Third, particular features were systematically undersampled. Fourth, two distinct model evolutionary pathways were simulated to generate synthetic observations reflecting a mixture of evolutionary dynamics. We found that observation noise levels up to 20% produced little qualitative change in the inferred dynamics (Figure S1), particularly in the more pertinent case of additional positives (modeling unobserved modes of tool use). Furthermore, systematic undersampling of a subset of modes still allowed the general dynamics to be recovered (Figure S2), and we found that HyperTraPS readily identified and could separate different evolutionary dynamics underlying different synthetic taxa (Figure S3). This robustness of inferred pathway structure to point perturbations in observations mirrors HyperTraPS behavior in other biological contexts (Williams et al., 2013, Johnston and Williams, 2016).

Two related points for investigation concern the taxonomic structure of our source data. The source data from Shumaker et al. (2011) samples some taxa (particularly primates) more densely than others. However, HyperTraPS naturally controls for heterogeneous sampling, appropriately weighting densely sampled small steps and sparsely sampled large steps to build an unbiased overall picture of the underlying dynamic pathways (Johnston and Williams, 2016), as we see in a focus on bird behavior (see below). Furthermore, there is a difference in levels at which observations are reported, which range from species to orders. The reporting of observations at higher taxonomic levels allows for some ambiguity in the data. For example, does an order wherein modes *A* and *B* are reported mean that a species within that order has acquired both *A* and *B*, or that two different species have, respectively, acquired *A* and *B* separately? HyperTraPS accounts for this ambiguity by, in the absence of further information, assigning all possible intermediate dynamics equal weighting (in this example, ∅→A,∅→B,A→A+B,B→A+B would be equally weighted). However, we wished to verify that this equal weighting did not contribute artifacts to our inferred posteriors that were not compatible with observations. In addition to the general approach above, where perturbations of up to 20% in the dataset did not lead to substantial inferred differences, we therefore also considered a rearrangement of the source data so that only observations resolved at the family level or below were included. We found good agreement with the posteriors from the original dataset (Figure S4), with the discrepancies explained by the omission of some taxa from the rearranged set.

Next, we asked whether different patterns of emergence were evident in behavior observed in the wild, compared with behaviors observed with some human influence. We note that Figure 1 clearly shows that *more* modes of behavior have been observed under human influence but does not show whether the acquisition of wild and human-influenced behaviors follow *different patterns*. Interestingly, the inferred emergence dynamics from exclusively wild observations did not dramatically differ from those inferred from all observations (Figure S5). The most notable differences were in the inferred ordering of *hang*, *bait*, *club*, *reach*, and *prop-climb*, all of which are observed more rarely in the wild (*hang* is observed exclusively under human influence) and thus have their ordering posteriors shifted to later times. Compensatory shifts occur in *cut*, *wave-shake*, and *block*, which show a shift in their late-ordering posterior density to earlier times. However, the overall structure of the inferred dynamics is remarkably comparable between cases. This consistency points to a picture where, rather than reshaping the dynamics of tool use acquisition, human influence simply facilitates more modes (for example, by making more manipulable objects available) and increased observation.

### Dynamically Identified Classes of Tool Use Complexity

The inferred dynamic pathways of emergence reveal four broad classes of tool use, running in decreasing order of acquisition propensity, labeled in Figure 2A. Class 1 modes have posterior ordering distributions weighted toward early acquisition times. Functionally, class 1 broadly corresponds to the simplest ways of moving objects in an environment. These include *affix*, *throw*, *reach*, and *drag-roll*. Basic exploitation of object-environment interactions (*bait* and *contain*) and the simplest possible modification of objects (*scratch*) also fall within this class.

Class 2 modes have posterior ordering distributions with more even weighting across orderings and are thus likely to occur at intermediate points on emergence pathways. Class 2 broadly involves more sophisticated ways of moving objects in an environment. These modes include *prop-climb*, *insert-probe*, and *drop*.

Class 3 modes have posterior ordering distributions skewed toward later acquisition times. Class 3 involves exploiting the physical properties of one object to achieve a goal involving another. These include *pound*, *club*, *absorb*, *wipe*, and *jab-stab*.

The final class 4 involves harnessing less simple, and even abstract, physical properties of objects to achieve a goal. These modes with posterior orderings strongly weighted to late acquisition, demonstrated exclusively by advanced primates, involve *hang* (invoking a three-dimensional object-environment interactions), *symbolize*, and *cut*.

Several modes, marked with asterisks in Figure 2A, do not fall naturally into these simple classes; instead, they display bimodal posterior distributions of acquisition ordering. Such bimodality is the signature of distinct evolutionary pathways in a system, where a feature may be acquired either early or late but not at intermediate orderings (Williams et al., 2013, Greenbury et al., 2019). These modes include *wave-shake*, *dig*, *block*, and *pry*, although limited bimodality can be observed in several of the modes above. Hence, distinct emergence pathways exist where, for example, *wave-shake* may be acquired either early or late in two different pathway types. We explore potential causes of these structurally distinct pathways below.

Taken together, these posterior orderings suggest a dynamic scheme for tool use emergence following intuitive concepts of complexity, potentially linked with stereotypical versus flexible modes (Hunt et al., 2013). The first modes to emerge involve simple manipulations of objects, then more sophisticated manipulations, followed by exploiting simple, and then more complex, properties of objects to achieve a goal involving a separate target.

### Environmental and Lineage Influence on Tool Use Emergence

The visible separation of distinct emergence pathways in Figure 2B suggests that multiple distinct dynamic pathways of tool use emergence exist. Given the observed bimodality in posterior ordering for several modes in Figure 2A, we next asked whether the acquisition patterns of particular tool use modes could explain this distinctive dynamic structure.

**Figure 3:**
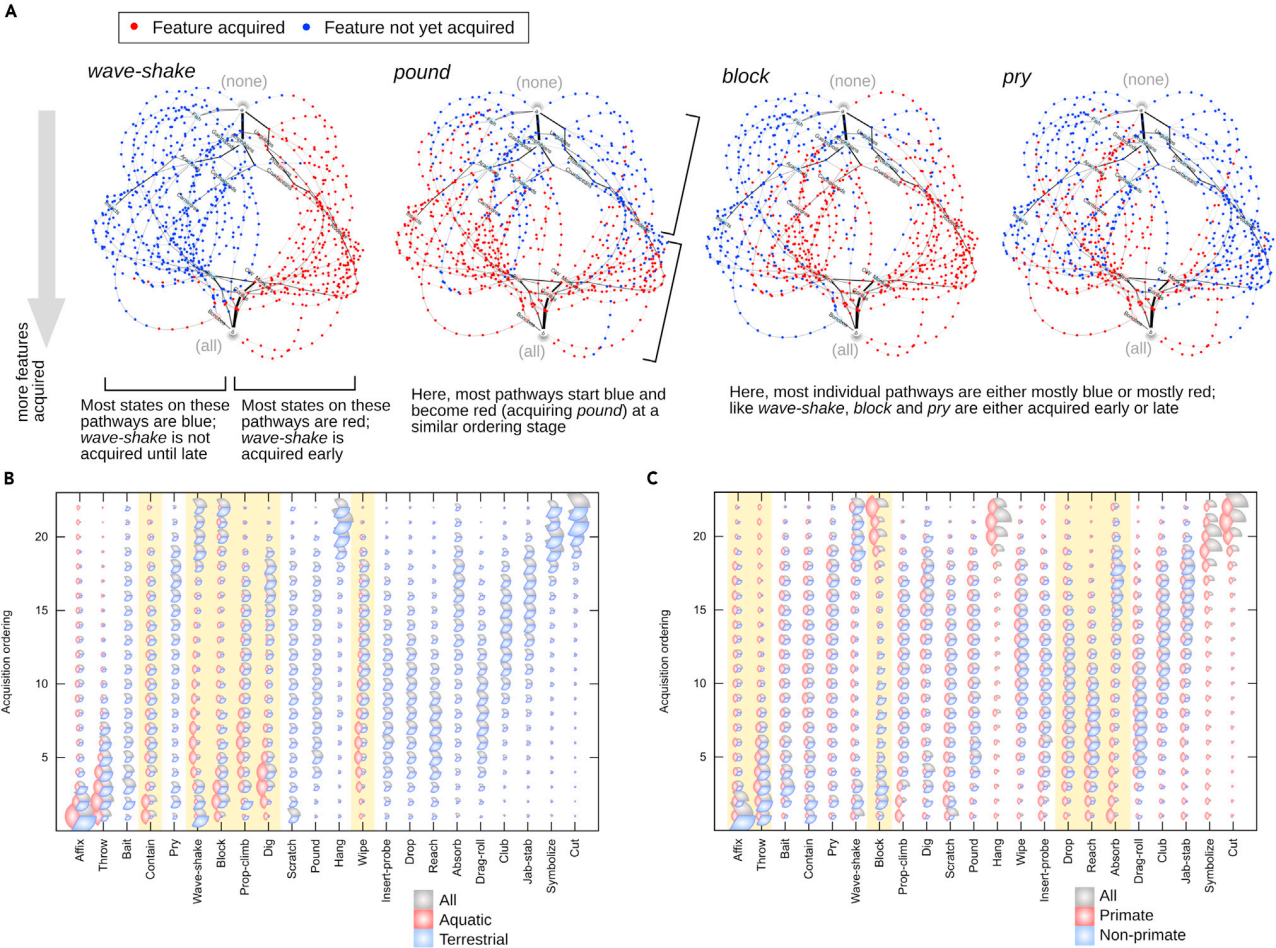
Structure and Predictors of Distinct Emergence Pathways (A) The high-probability pathways from Figure 2, with states labeled according to the presence or absence of specific modes. Acquisition of *wave-shake* distinguishes pathways in different regions of the space (here left, where *wave-shake* is acquired late, and right, where it is acquired early). *Pound*, as a counterexample, does not separate regions of space to the same extent, being acquired at a similar stage in pathways in all regions. *Block* and *pry* also display different acquisition orders in different regions. (B and C) Posterior orderings inferred using subsets of the data. Gray (top right) arcs give the posterior probabilities inferred from the full dataset (Figure 2); red and blue arcs give posteriors inferred using the two subsets. Some subsets have no observations of some modes: in these cases, the uniform prior is recovered, which is not plotted here. Specific modes, discussed in the text, displaying posterior differences between subsets are highlighted. (B) Aquatic and non-aquatic subsets; (C) primate and non-primate subsets.

Figure 3A shows instances where different tool use modes have been acquired through the distinct emergence pathways found in Figure 2B. We immediately observe that the two sets of pathways making up the “left” and “right” regions in the hypercube embedding are almost perfectly distinguished by the presence or absence of *wave-shake* and *block*. One set of pathways, enriched for non-primate groups, involves the early acquisition of *block*; the other set, enriched for primate species, does not acquire *block* until nearly all other modes have been acquired. *block* is indeed observed across a range of non-primate animals, reflecting, for example, octopuses blocking oyster shells open with coral (Lane, 1957), rooks blocking drainage holes with plugs (Reid, 1982), elephants blocking previously dug water holes to prevent rival animals using them (Gordon, 1966), and species including ground squirrels, beavers, badgers, and naked mole rats blocking tunnels and burrows (McLean, 1978, Aeschbacher and Pilleri, 1983, Shuster and Sherman, 1998, Michener, 2004).

The two distinct branching pathways in the hypercubic network are then distinguished by the presence of *wave-shake* and, to a slightly lesser extent, *pry*. One pathway (occupied by amphibians, gastropods, cephalopods, birds) involves the early acquisition of *pry* but not *wave-shake*; the other (occupied by crustaceans, rodents, prosimians, elephants) involves early acquisition of *wave-shake* but not *pry*. The first of these pathways reflects, for example, birds using tools to pry out insects from holes, bark from trees, seeds from feeders, and even to pry apart human toes in play (Shumaker et al., 2011, Merlen and Davis-Merlen, 2000); the second reflects examples including boxer crabs waving captive anemones for defense and food capture (Bürger, 1903), lemurs waving scent at rivals for intimidation (Jolly, 1966), and elephants “fly switching” with waved branches (cited in Shumaker et al. [2011] as perhaps the first documentation of animal tool use, by Harris [1838]). These two branching pathways further disperse through the *block*-separated regions above.

Each of these pathway-differentiating modes displays pronounced bimodality in its corresponding ordering posterior (Figure 2A), illustrating the correspondence between bimodality and distinct pathway structures. We next asked whether this bimodality could be explained by different emergence dynamics experienced by animal groups from different lineages, or in different environments (Visalberghi et al., 2005).

We first considered environmental influences and studied perhaps the largest environmental difference in our dataset: aquatic versus terrestrial taxa. To examine environmental influence on tool use emergence, we split the original dataset into two subsets, corresponding to aquatic and terrestrial taxa. We performed HyperTraPS inference with both these subsets independently, and compared the resulting posteriors with each other and with that from the original superset. Several notable differences between the umbrella inference and the aquatic subset are immediately observed (Figure 3B). Aquatic taxa have moderately early acquisition orderings for *wave-shake* (found above to distinguish emergence pathways on the hypercube) and for *contain* and *dig* (which also displays some bimodality above). These modes reflect, for example, the aforementioned crabs waving anemones (Bürger, 1903), octopuses excavating shelters with water jets (Mather, 1995), and dolphins using sponges while digging for fish (Krützen et al., 2005). Aquatic taxa also show a comparatively early acquisition of *wipe*, reflecting, for example, the observed behavior of captive dolphins wiping their aquarium windows, with a feather (Tayler and Saayman, 1973). When considering only behavior observed in the wild, the aquatic-land discrepancy in *wave-shake* is somewhat reduced, but other trends remain clear (Figure S6); a later acquisition of *drop* in aquatic organisms is also apparent. These discrepancies are mirrored in the inferred posteriors for non-aquatic taxa.

We next compared primate and non-primate taxa (Figure 3C). In primates, a more uniform ordering of several early modes including *affix* and *throw* is observed. *block* is notably acquired comparatively late among primates, whereas several otherwise later modes (including *drop*, *reach*, *absorb*) are acquired earlier. Correspondingly, in the non-primate case, early class 1 modes like *affix* and *throw* have an even higher early weighting, whereas class 4 modes are not observed at all. These trends remain robust when only behaviors observed in the wild are considered (Figure S6), reflecting the point above that the number, but not necessarily the patterns, of modes of primate tool use are different in the wild.

Shumaker et al. (2011) provides a further detailed analysis of tool use in the next most tool-using taxon, birds. To examine emergence patterns at a different taxonomic scale, we used these data to perform a similar analysis of pathway inference using data only from bird species (Figure S7). Comparing avian tool use emergence with other species (Figure S7) highlighted the early emergence of avian modes including *pry*, *dig*, *insert-probe*, and *jab-stab*, and late emergence of *throw*, intuitively linking with avian physiology. *bait* also displayed a relatively early acquisition propensity.

Taken together, these results suggest that the distinct pathways resulting from splitting bimodal *wave-shake* (and *dig*) distributions reflect different emergence dynamics in aquatic and non-aquatic environments. Aquatic organisms acquire these modes earlier, whereas some non-aquatic organisms acquire them late (if at all). The distinct pathways resulting from splitting *block* seem to correlate in part with primate versus non-primate taxa. Furthermore, the more limited bimodality associated with *pry* may in part be explained by its early acquisition in birds compared with other taxa (Figure S7). These splits differentiate the broadest classes of pathways on the hypercube (Figure 3A). The inferred acquisition ordering of several other modes of tool use appear quite consistently across environments (for example, early acquisition of *affix* in both aquatic and non-aquatic cases), suggesting similarities in the acquisition of tool use across environments and families (see Discussion).

### Prediction of Future and Unobserved Behaviors

The Bayesian nature of HyperTraPS means that predictions, with quantified uncertainty, can be made about the behavior of the underlying dynamic system. First, we asked which modes in which groups may correspond to extant, but presently unobserved, tool use characteristics. To this end, we simulated pathways on the inferred hypercube and recorded every time a state was encountered that had the same pattern of observed modes as an animal group (i.e., the same pattern of presence markers, but not necessarily the same pattern of absence markers). For each of these compatible states, we recorded which of the other modes were present and which were absent, iterating over many pathways to obtain the posterior probabilities with which each unobserved mode was present or absent in states that could correspond to each observation.

More speculatively, we also asked which tool use modes may be expected to emerge next in the future, given the inferred pathways of tool use emergence across taxa. To this end, we simulated evolution on the inferred hypercubic posteriors, and every time a state corresponding to a real observation was encountered (i.e., those states highlighted in Figure 2B), we recorded which mode was next acquired in that simulation. Over many simulations, we thus obtain the probability associated with each mode being acquired next from each observed state.

**Figure 4:**
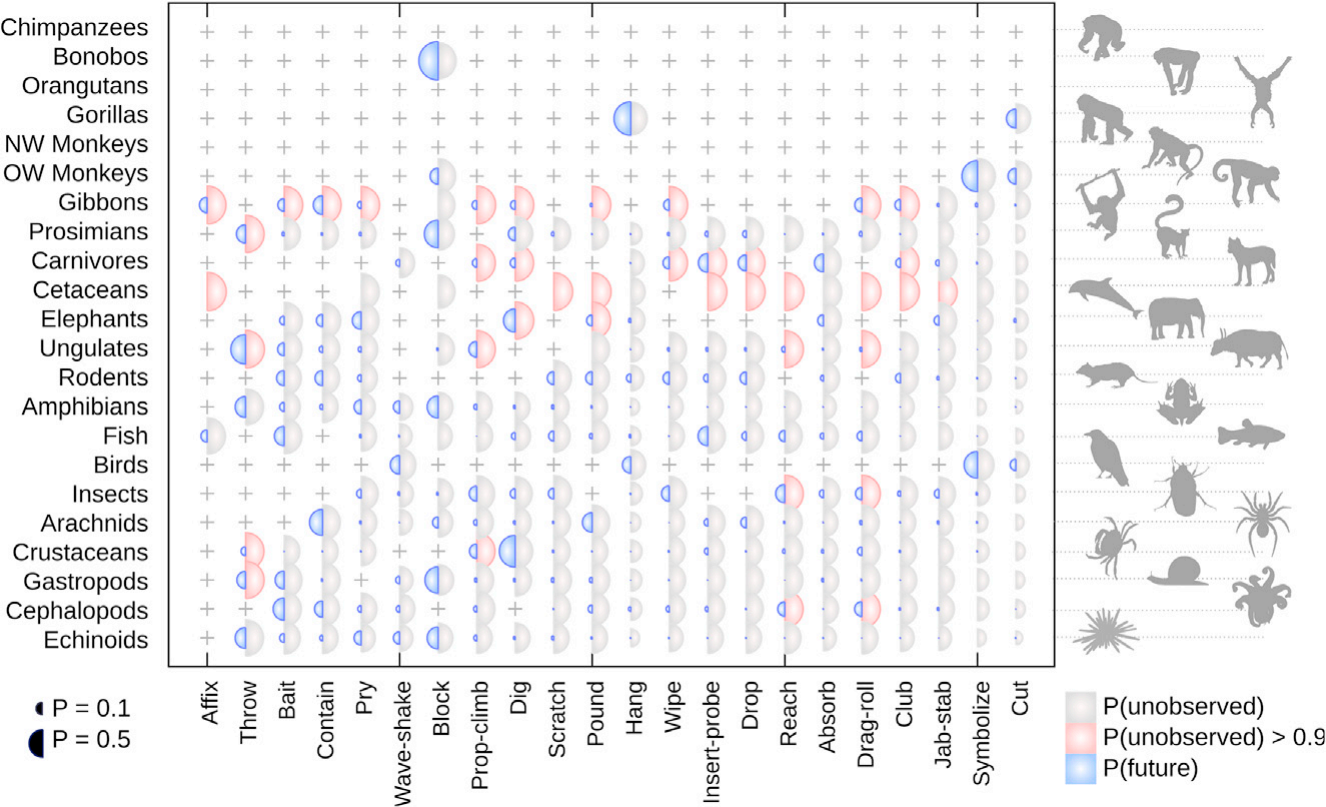
Prediction of Unobserved and Future Behavior Crosses mark those modes (horizontal axis) already observed in given groups (vertical axis). Red-gray: potential unobserved modes of tool use. With prior belief that all negative observations are equally likely to represent present but unobserved modes of tool use, circles give the posterior probability that a given mode is present but unobserved. Red circles denote a posterior probability over 90%. Blue: potential future emergence of tool use. Assuming that negative observations reflect genuine absences, circles give the posterior probability that a given mode will be the next to be observed in a given group. NW, New World; OW, Old World.

Figure 4 shows the resulting probabilities. Notable pathway dependence is visible in these predictions. Intuitively, modes that are generally acquired early (like *affix* and *throw*) are assigned a high probability of being present in those groups where they remain unobserved. Several class 1 and class 2 modes are assigned a high presence probability in cetaceans and ungulates; probabilities across other groups are generally lower. We also see pathway dependence in future acquisition predictions, observing, for example, a reasonable probability for class 1 *affix* in gibbons, but not for fish, which have proceeded down a different pathway (linked to their aquatic environment) and are thus inferred to be more likely to acquire other modes first. For the same reason, some class 2 and class 3 modes (for example, *drop* and *club*) are likely to be acquired next by groups that have not acquired many other modes, potentially once more linked to the implicitly learned *stereotypical* versus *flexible* distinction in tool use acquisition (see Discussion).

## Discussion

We have used HyperTraPS, an approach for the inference of evolutionary pathways, to characterize the dynamic pathways by which observed patterns of tool use have emerged across animal taxa. The structures of these pathways are strikingly conserved across animals, and their posterior orderings reveal different classes of tool use mode, with decreasing propensity linked to a qualitative theme of increasing complexity. Modes involving the simplest manipulations of objects are highly likely to be acquired early; modes involving more sophisticated interactions between objects and individuals are likely to be acquired later. Several structurally distinct pathways of emergence are readily identified by HyperTraPS, distinguished largely by one or two modes; these distinct pathways are linked to specific physical environments (aquatic or non-aquatic) and lineages (primates or non-primates).

Given the taxonomic diversity of species considered here, it is notable that these pathway structures appear with some consistency across taxa. Certainly the observation dataset (Figure 1) displays some structure, but the canalization of the branching emergence pathways revealed by HyperTraPS (Figure 2) is rather more striking than in previous biological questions studied with this method (Johnston and Williams, 2016, Williams et al., 2013). Several of the most pronounced differences between the different canalized pathways are linked to the environment or ecological niche that a species occupies (Figure 3), as expected from studies within species (Shumaker et al., 2011, Fujii et al., 2015, Humle and Matsuzawa, 2001). Here, these different pathways are typically distinguished by differences in the acquisition ordering of a relatively small number of modes, which display bimodal distributions of acquisition ordering (for example, *wave-shake* and *block*). Many other modes display rather consistent acquisition ordering across taxa and environments. This consistency suggests that similar principles may apply to the emergence of tool use across a wide range of taxa and environments, and that the acquisition dynamics of different modes have a substantial component of convergency. Furthermore, the presence of some similarities between the emergence pathways inferred across taxa, those inferred from family-level observations (Figure S4), and those inferred using exclusively avian observations (Figure S7) suggest a scale-free structure to our results that holds across different phylogenetic levels. We hope to demonstrate that more cross-taxa comparative study of tool use emergence has the potential to shed light on these general principles.

Of course, there is substantial debate and flexibility in what constitutes tool use, or a specific mode within that class (Hunt et al., 2013, Shumaker et al., 2011, St Amant and Horton, 2008). The source of our dataset, for example, does not insist upon dynamic mechanical interactions, which some literature considers as an essential part of tool use (Hunt et al., 2013, St Amant and Horton, 2008). Different modes are often considered (for example, *cleave* as an intermediate between *pound* and *cut* [St Amant and Horton, 2008]). To account for this flexibility, we performed inference using a selection of perturbed datasets, reflecting variable definitions and observations, and found that the pathway trends we observed were robust to these perturbations. Beck (1980) and Shumaker et al. (2011) consider a set of broad functional classes of tool mode use: extending the user's reach, amplifying force, augmenting display, controlling fluids or small objects, and comfort. However, we found a stronger link between our “complexity”-based classes and the emergence dynamics we observe.

We underline that complexity in biology, and particularly in behavior, is a highly nuanced topic (Schuster, 1996, McShea, 1991, Sambrook and Whiten, 1997). It is not clear that the concept of a quantitative definition of complexity is even well posed in many instances, and such a quantitative definition is a prerequisite for any robust statistical analysis. Rather than claiming any direct link to a given measure of complexity, we quantitatively report the inferred structure of the pathways we observe and simply note that the dynamic classes that emerge align with an intuitive, qualitative concept of complexity. This intuitive link could be more formally explored in future by, for example, using an appropriate measure of complexity to compare the cognigrams associated with different modes of tool use emergence (Haidle, 2010, Hunt et al., 2013). In addition, this notion of complexity in tool use may not be unrelated to that of *stereotypical* versus *flexible* tool use (Hunt et al., 2013). Stereotypical tool use is characterized by what is likely a minor adaptation of non-tool use behavior, is limited to a specific scenario, and is widespread or fixed in a given species (bearing a greater resemblance to instinctive rather than learned behavior). This is the predominant tool use type in taxa where tool use is rare (for example, insects and gastropods). In contrast, flexible tool use is more adaptable to different circumstances, can be patchy in terms of intraspecific distribution, is likely perpetuated by learning from conspecifics, and probably requires some level of prior conception of the external problem to be addressed by the tool. This category is mostly limited to primates, and, to a lesser extent, to birds such as New Caledonian crows, plausibly evolving late and requiring a greater degree of working memory capacity.

### Limitations of the Study

Substantial work exists using model-driven approaches to explore the emergence of tool use (Hunt et al., 2013). We do not intend to compete with this wide and expert literature; rather, we intend our data-driven approach to complement these more mechanistic considerations. By providing a platform where current observed evidence can be unified and trends identified, we hope to provide a valuable comparative approach to support and refine mechanistic models.

In seeking breadth, we have used a catalog of available data at varying taxonomic levels across animals. As discussed above, these differing levels mean that we lack species-specific dynamic information in some cases and must instead assign equal probabilities across a set of different possible transitions. These data also omit the finer-grained dynamics of tool use acquisition in individual animals, instead reporting the capacity for a species to develop a particular mode of tool use. The possibilities of convergent evolution and human influences on these observations must also be borne in mind. We have attempted to quantify the impact of these various features and possibilities on our results, generally finding that our results remain robust across these possibilities.

HyperTraPS also facilitates the prediction of unobserved and future behaviors. As this is a purely data-driven approach, functional considerations are not taken into account and some predictions may therefore be unfeasible: it is perhaps hard to imagine echinoderms employing *throw*, for instance (Figure 4A). However, the Bayesian nature of these predictions means that functional considerations can readily be taken into account by adjusting the prior belief associated with each of these predicted modes. Expert knowledge may be used to limit or remove prior support for particular modes, and the posterior predictions will be naturally adjusted accordingly. On a related note, as our HyperTraPS approach does not account for reversibility (traits are modeled as irreversibly acquired once “discovered”), further observational data will be required to characterize the loss of tool use traits if they occur. More broadly, further and more detailed observations will always help refine the inferred posteriors and associated theory: we hope to demonstrate that HyperTraPS provides a useful platform to integrate and harness more detailed future observations and that such ongoing observation of animal behavior can be used to test and refine the picture developed here.

### Resource Availability

#### Lead Contact

Further information and requests for resources and reagents should be directed to and will be fulfilled by the Lead Contact, Iain Johnston (iain.johnston@uib.no).

#### Materials Availability

This study did not generate new materials.

#### Data and Code Availability

All data and code for this project are freely available on Github at github.com/StochasticBiology/tool-use.

## Methods

All methods can be found in the accompanying Transparent Methods supplemental file.

## Supplemental Information

Document S1. Transparent Methods, Figures S1–S6, and Table S1
