# Supplementary material for: Data-Driven Inference Reveals Distinct and Conserved Dynamic Pathways of Tool Use Emergence across Animal Taxa
Source: iScience. 2020 Jun 9;23(6):101245. doi: 10.1016/j.isci.2020.101245 (PMC7306607; doi:10.1016/j.isci.2020.101245)
Supplement: Document S1. Transparent Methods, Figures S1–S6, and Table S1 [file mmc1.pdf]

iScience, Volume *23*

Supplemental Information

Data-Driven Inference Reveals Distinct and Conserved Dynamic Pathways of Tool Use Emergence across Animal Taxa

Iain G. Johnston and Ellen C. Røyrvik

Supplementary Figures

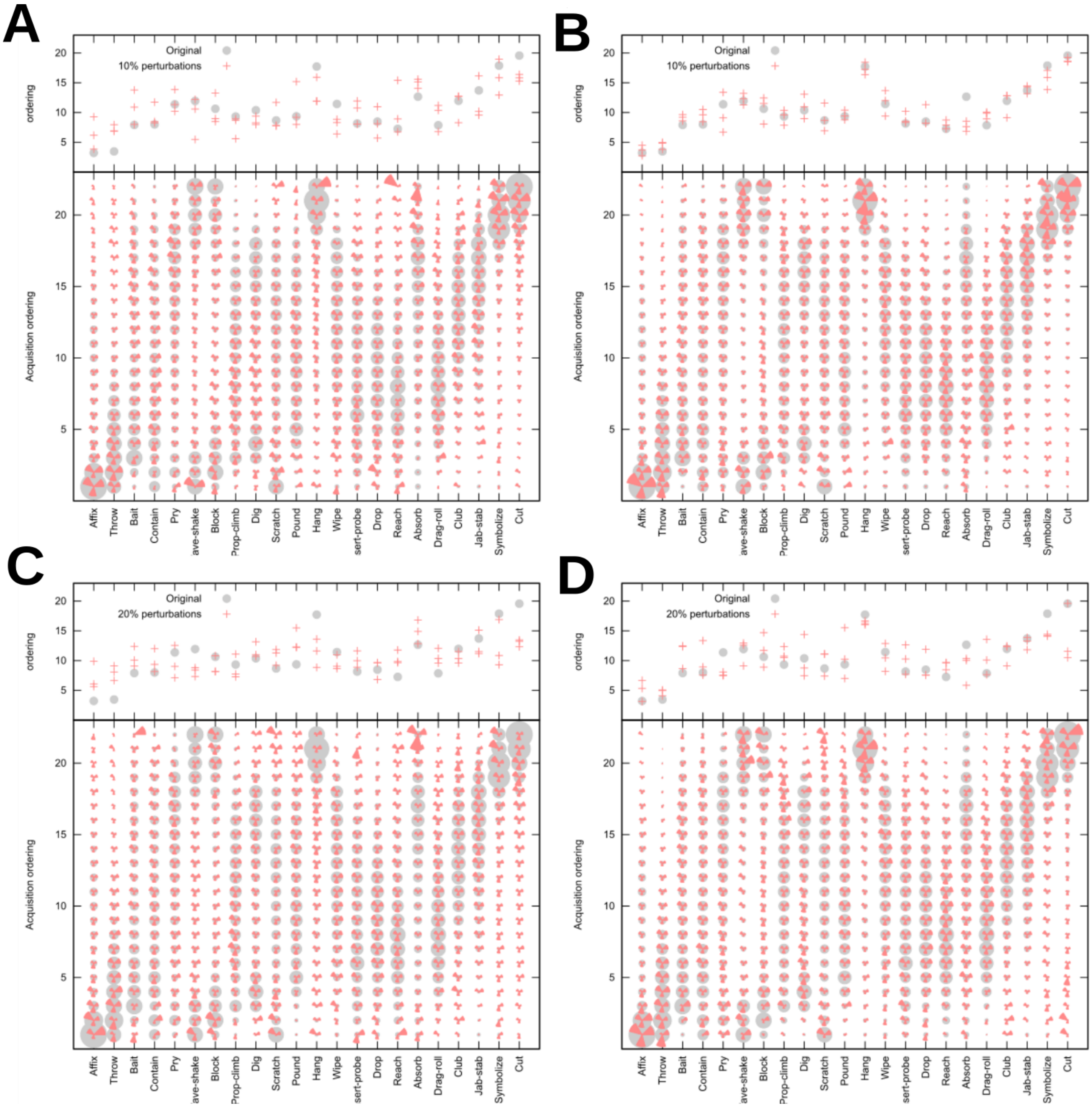

Figure S1: **Robustness of inferred posteriors to observation variability (related to Fig. 2).** Grey circles give posteriors inferred using original dataset; red segments give posteriors inferred using three different synthetically perturbed datasets. Single points in upper plots show means of inferred posteriors. (A) General observation noise: both presence and absence observations are randomly perturbed with probability 10% (B) Occluded observations: absence observations are changed to presence observations with probability 10%. (C-D) As (A-B), but with perturbation probability 20%.

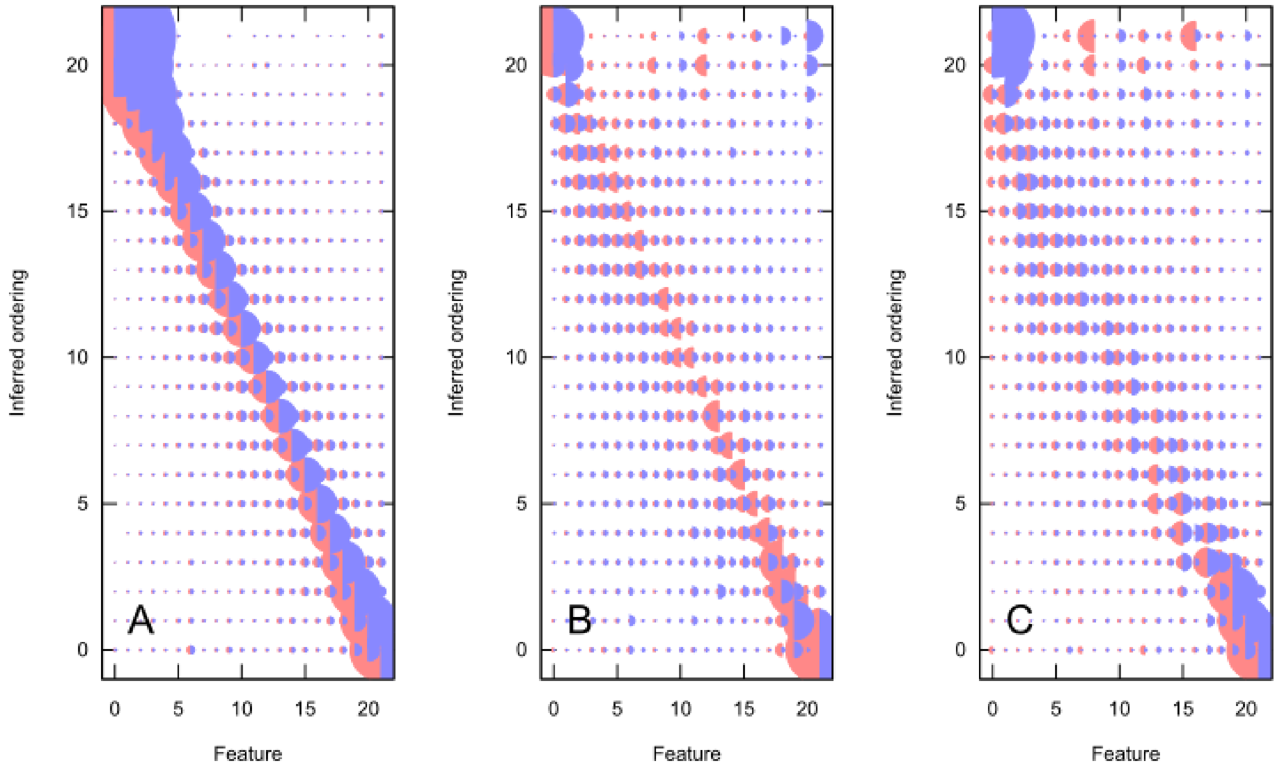

Figure S2: **Effects of systematic undersampling (related to Fig. 2).** Synthetic experiments with 22 modes and 22 observations, modelling the data used in this study. Two randomly generated datasets (red and blue) are used. (A) Original synthetic dataset supporting a single evolutionary pathway. (B) Even-numbered modes are systematically undersampled by 10%. (C) Even-numbered modes are systematically undersampled by 20%.

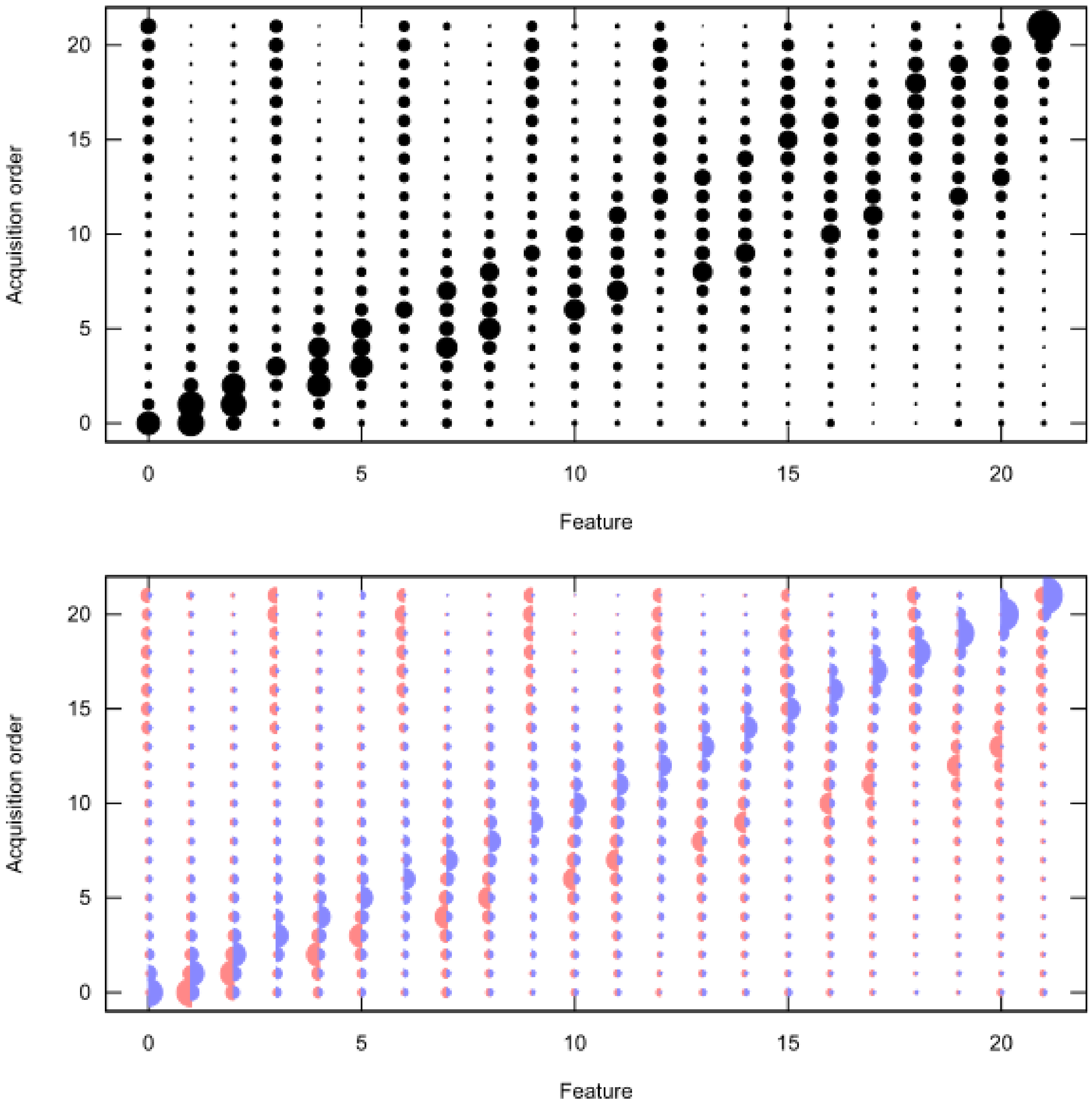

Figure S3: **Detecting different evolutionary pathways (related to Fig. 3).** Two synthetic evolutionary pathways are used to generate these data, one involving (i) simple one-by-one acquisitions from the first to the last feature, and one involving (ii) acquisitions from first to last except every third feature. One class of 'organism' is simulated for each pathway. (A) The inferred posterior orderings for the amalgamated dataset of both classes together: the different pathways give rise to bimodality in the overall posteriors. (B) Inferred posterior orderings for the two classes analysed separately. The different evolutionary dynamics (blue (i) and red (ii)) are readily identified and separated, and their contributions to the amalgamated posterior clearly observed.

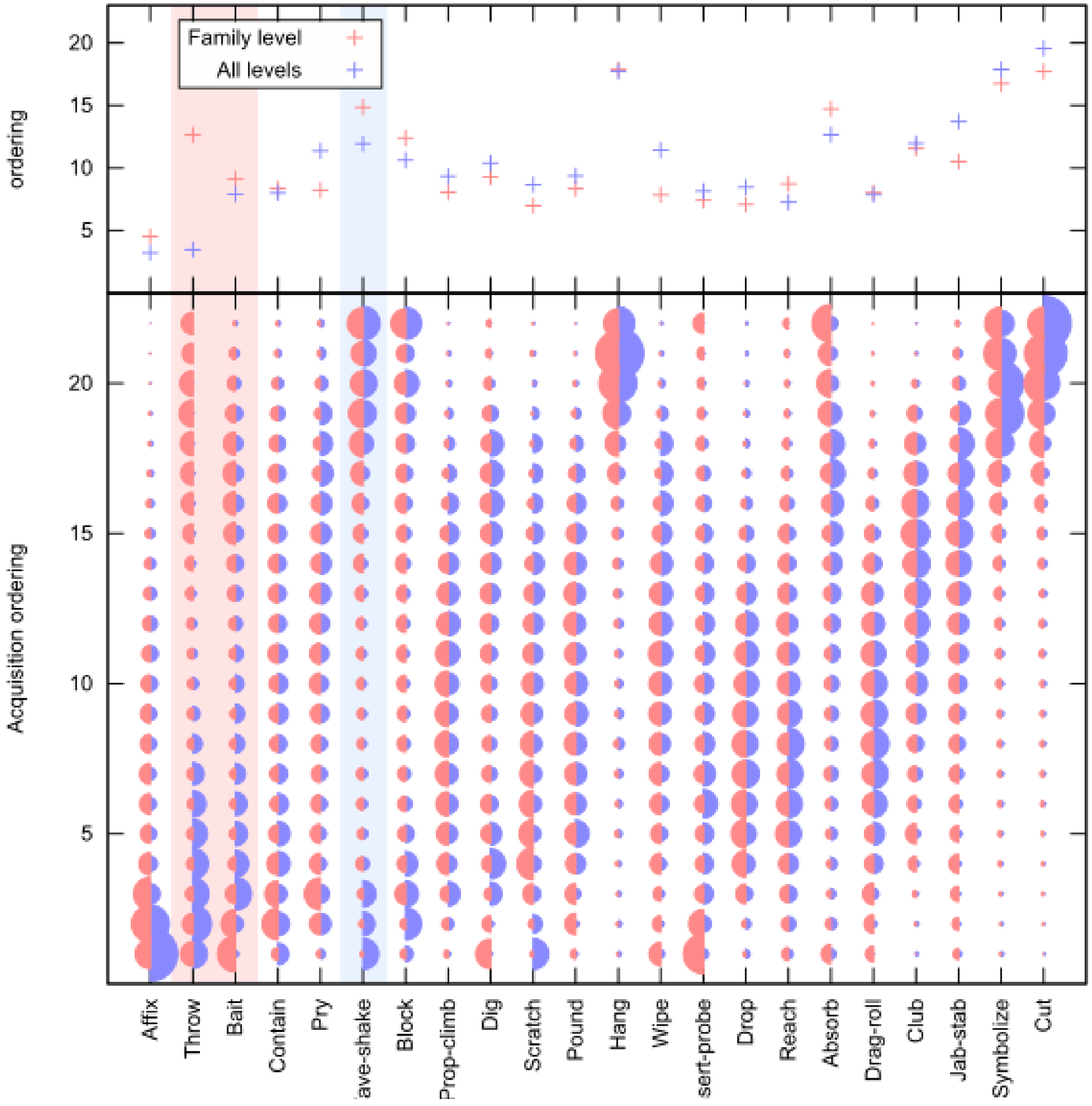

Figure S4: **Inferred posteriors for observations restricted to be unambiguous at the family level (related to Fig. 2).** Blue posteriors are from the original inference process; red posteriors are those from a rearranged dataset involving only family-level (or otherwise unambiguous) observations. Single points in upper plots show means of inferred posteriors. General structures are similar; *throw* and *bait* (highlighted red) display more bimodality in the family-level data, and *wave-shake* (highlighted blue) displays more bimodality in the original data. These differences largely reflect taxa omitted from the family-level data. For example, insects, arachnids, and cephalopods display *throw* and few other modes, suggesting it to be an earlier acquisition, but these groups are omitted from the family-level inference, pushing the mean inferred acquisition ordering to later times.

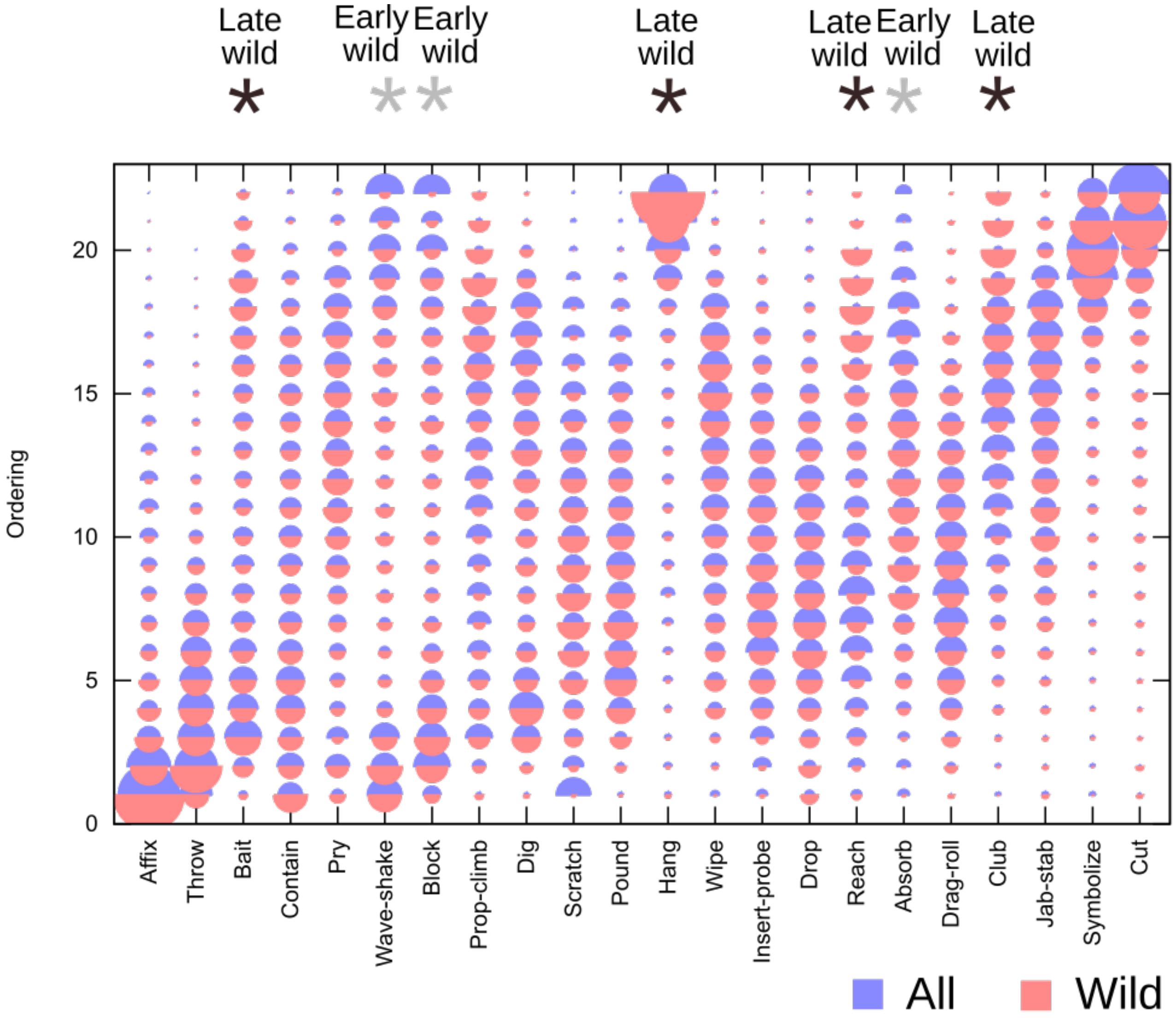

Figure S5: **Robustness of inferred posteriors to wild, versus human-influenced, behavioural observations (related to Fig. 2).** Blue posteriors give emergence pattern inferred from all observations; red posteriors show those inferred using only observations of behaviour in wild animals. Some modes are comparatively rarely observed in the wild (especially *hang*, only observed under human influence) and these posteriors are shifted to marginally later times (black stars). Compensatory shifts (grey stars) occur in some other modes. However, the patterns of emergence remain comparable for both datasets.

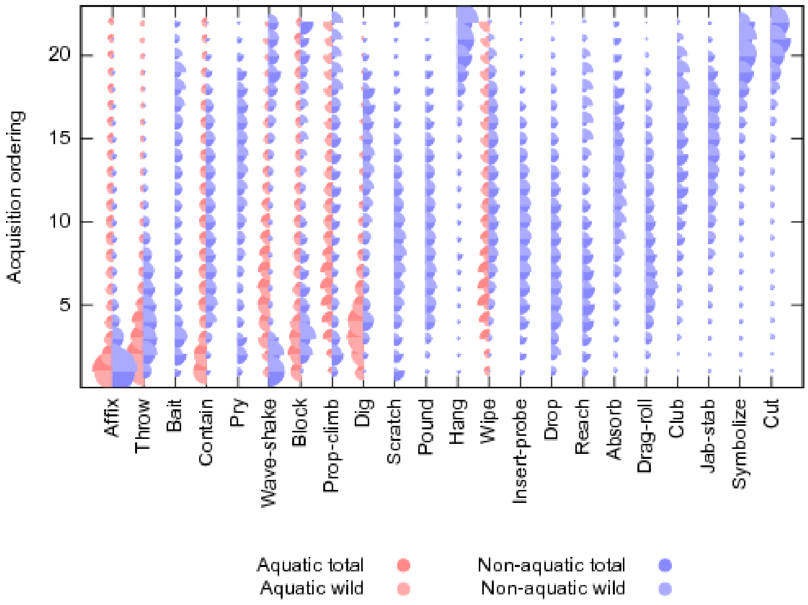

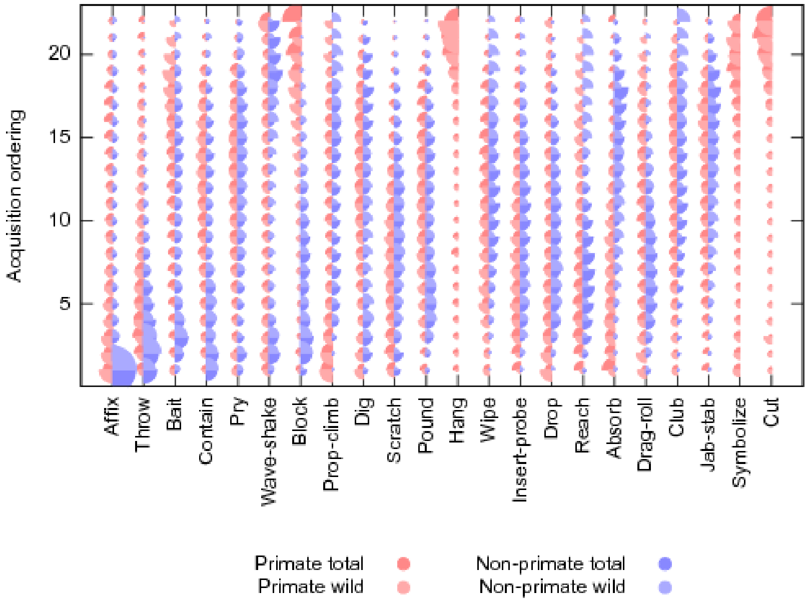

Figure S6: **Environmental and lineage correlates using only wild behavioural observations (related to Fig. 3).** (A) Aquatic-terrestrial and (B) primate-nonprimate comparisons of emergence patterns. Darker posteriors ('all') are those in the main text, inferred from the full set of behavioural observations. Lighter posteriors ('wild') are those inferred using only behavioural observations in wild animals. Most differences between the classes in (A) and (B) remain comparable in both cases (for example, *block* is acquired later in primates than non-primates for both datasets). Departures from these patterns are discussed in the main text.

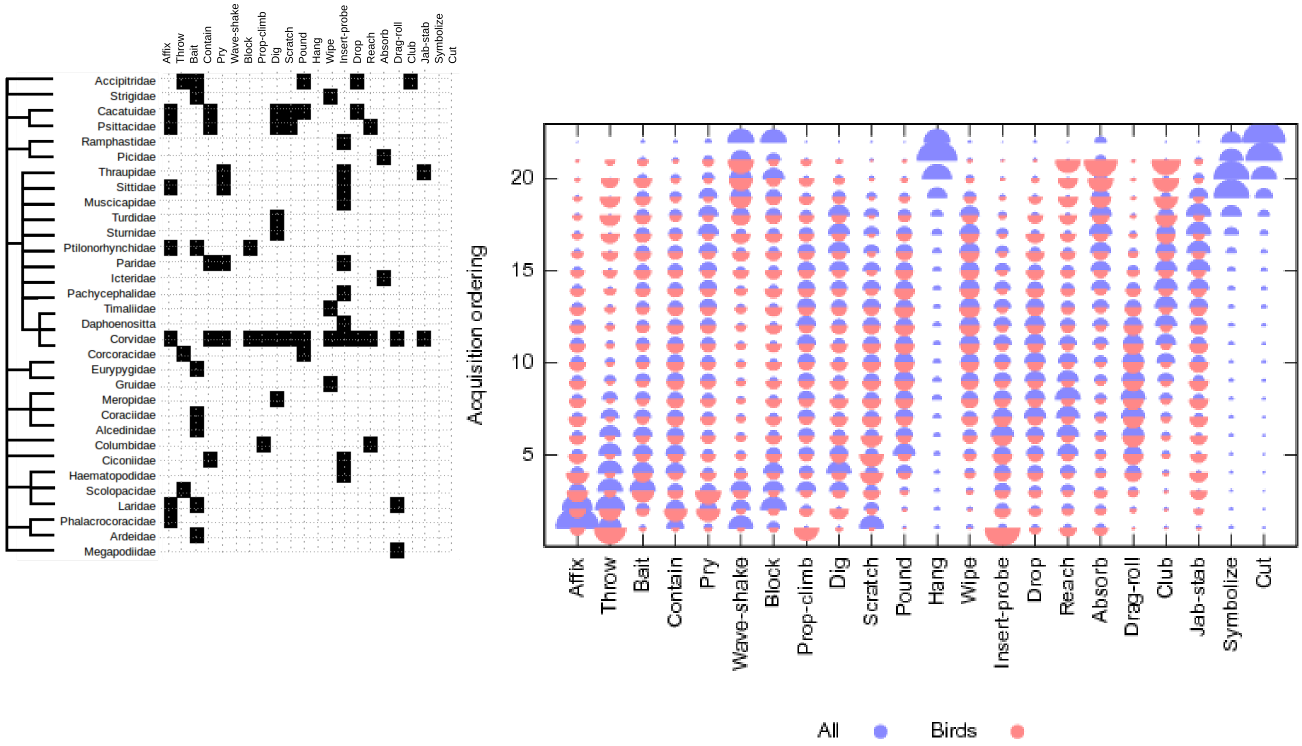

Figure S7: **Emergence patterns of tool use modes in birds and other species (related to Fig. 2).** (left) Data on tool use in different bird species, after Shumaker et al. (2011). As in the original dataset, *symbolize* is missing from this set of observations (no birds display this mode). (right) Inferred posteriors from bird tool use. Blue posteriors give emergence pattern inferred from all observations; red posteriors show those inferred using only observations of behaviour in birds.

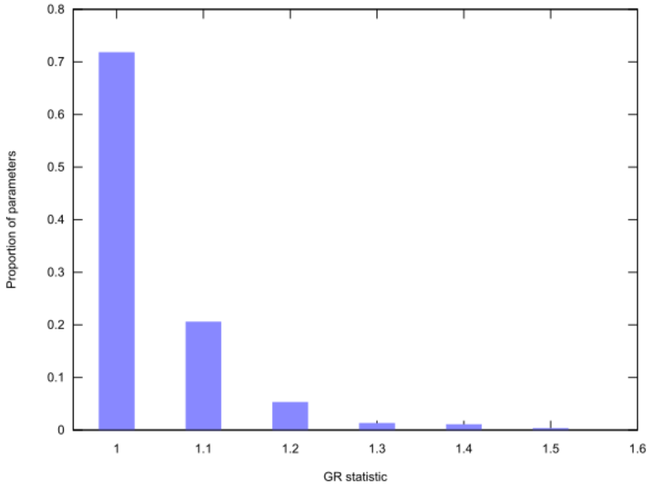

Figure S8: **Convergence of MCMC chains (related to Fig. 2).** The Gelman-Rubin diagnostic (Gelman et al., 1992) for the MCMC chains in our inference process. Values are calculated for each parameter in the model and counts are binned. Values of 1 is the limit of perfect convergence; values under 1.1 are often interpreted as good evidence for convergence.

Supplementary Tables

Drop	Cause an object to fall. The object is propelled primarily by gravity.
Throw	Propel an object through open space. Can be aimed or unaimed. The object is propelled by the user's own energy.
Drag	Pull an object while walking or running; the object touches the substrate.
Roll	Propel an object such that it turns over repeatedly on the substrate.
Kick	Propel an object with foot through space or on the substrate.
Slap	Hit an object or another organism with a flattened appendage.
Push over	Deflect an attached vertical object.
Brandish	Conspicuously display an object away from the body.
Wave	Move an unattached object repeatedly up and down or side to side.
Shake	As in Wave, but the object is attached.
Bait, Entice	Manipulate or place, and conspicuously display, an object such that it attracts prey or a potential interactant to approach.
Club, Beat	Hit another organism antagonistically with an object. The object (the tool) can be another organism or a part thereof.
Pound, Hammer	Hit an object or prey item forcefully, often repeatedly, with a second, relatively hard object (the tool).
Pry, Apply Leverage	Push or pull on an object (the tool), using a fulcrum.
Dig	Excavate the substrate, usually earth.
Jab	Use the distal end of an object to forcefully push another object or organism away, or to animate another organism; synonymous with prod.
Stab	Use the distal end of an elongate object to forcefully puncture or perforate another organism so as to harm it.
Penetrate	Use the distal end of an object, usually elongate, to forcefully puncture or perforate a surface or an inanimate object. Often followd by Insert and Probe. May be combined with Reach to allow retrieval of the object.
Reach	Use an elongate object to touch or retrieve another object when the user's prehensive structures are too short, or use objects of any shape to avoid touching the target object. Tool held throughout use. Can be followed by Scratch or Rub when affected body surfaces are inaccessible.
Insert, Probe	As in Reach, but when the target object is embedded in a hole or behind a restricted opening. Can include Insertion in bodily orifices.
Scratch, Rub	Move an object across a bodily surface, often repeatedly, while applying pressure. Includes tickling. Can follow Reach, if a body surface is inaccessible without the tool. Can be combined with Insert and Probe within a bodily orifice.
Cut	Movement, usually repeated and back and forth, of an object with a sharp edge to incise another object or surface.
Block	Place an object to prevent or impede movement or action of another object, fluid, or organism.
Prop, climb	Place and stabilise an elongate object vertically or diagonally against another object or surface, and then move up or climb up the object. Distal end of propped object touches the other object or surface. Stable.
Balance, climb	Place an elongate object vertically and then move up or climb the object. The distal end of the balanced object does not touch another object or surface. Unstable.
Bridge	Place an elongate object or organism over water or open space such that each end rests on a surface on opposite sides of the water or spatial gap. User locomotes on the object. Stable.
Reposition	Relocate and climb on an object or organisms. Includes rafting (placing a buoyant object on water to support user's weight).
Hang	Suspend the object(s) from an overhead substrate or surface. User locomotes or rests on the object.
Contain	Place fluids or objects into or on top of another object (the tool) to control and/or transport them.
Absorb	Soak up and move fluid. May be combined with Wipe.
Wipe	Mechanically displace and remove fluid, soft solids, or small solids. May be combined with Absorb.
Affix	Attach an object to the body, a surface, or another organism with adhesive.
Apply	Attach a fluid or an object to the body, a surface, or another organism without adhesive.
Drape	Place an object on the body or on a surface temporarily.

Table S1: **Definitions of different modes of tool use (related to Figs. 1-4).** Taken from Shumaker et al. (2011).

Transparent Methods

All data and code for this project are freely available on Github at `github.com/StochasticBiology/tool-use`.

Data

The catalogue we use defines tool use as "the external employment of an unattached or manipulable attached environmental object to alter more efficiently the form, position, or condition of another object, another organism, or the user itself, when the user holds and directly manipulates the tool using or prior to use and is responsible for the proper and effective orientation of the tool" (Shumaker et al., 2011). It identifies 22 different modes of tool use, given in figures throughout the article and described in Table S1. We manually labelled the data for wild versus human-influenced observations based on the qualitative description of each observation.

HyperTraPS

We consider the set of all possible states consists of every possible pattern of presence or absence for each of our $L = 22$ modes of tool use. The progressive acquisition of modes of tool use is modelled as a pathway through this space, moving from less recent to more recent states. For example, the first step on a pathway may involve a move from an initial state of *000...* (no modes of tool use acquired) to the next state *100...* (only the first mode, *affix*, acquired). We only allow transitions corresponding to the acquisition of exactly one mode at a time.

The model underlying HyperTraPS uses a set of parameters to describe the relative probabilities of acquiring traits (here, modes of tool use) in any given state (here, existing patterns of tool use modes). For example, under one parameterisation, in state *000...* we may have a probability of 0.4 of moving to state *100...* as in the example pathway above. There may be a probability of 0.2 of moving to state *010...*, 0.1 for moving to *001...*, and other possibilities. The product of the probabilities corresponding to the patterns of tool use observed in a dataset give the likelihood associated with a given parameterisation (Johnston and Williams, 2016; Greenbury et al., 2019).

HyperTraPS simulates a set random walkers travelling on these probabilistic pathways to estimate the likelihood associated with a set of observations for a given parameterisation. We then use MCMC to explore the space of possible parameterisations and, given some uninformative prior belief about these parameters, to build up a posterior distribution describing our belief about these parameters given a set of observations (Johnston and Williams, 2016; Greenbury et al., 2019). This posterior distribution reflects the parameterisations of the model that are most compatible with our data. Typically, the absolute values of these parameters are not of central interest, but rather we interrogate the different behaviours that these parameter sets support. This is the approach we use to build up the posterior plots of orderings and pathways in the main text (see below).

Here, we use the L^2 parameterisation scheme for HyperTraPS described in (Johnston and Williams, 2016), allowing the possibility of every mode's acquisition independently influencing the acquisition propensity of every other (although such influence is not assumed, and can be zero). This scheme represents individual transition rates as $P(\text{gain}\, i | \text{state}\, s) \propto \exp(\theta_{ii} + \sum_{j \neq i} \theta_{ij} s_j)$. Here, θ_{ii} is the logarithm of a basal rate of acquiring mode i, and θ_{ij} describes the multiplicative influence of s_j (the presence of mode j in current state s) on this basal rate for mode i. These rate parameters are used in constructing possible orderings of feature acquisitions for comparison with data, but their absolute values do not here correspond to experimental observables. Our subsequent analysis focusses on the sets of behaviours supported by observations, rather than the specific values of these parameters.

HyperTraPS is a Bayesian approach, allowing prior information and assumptions to be naturally included in the analysis of data. However, in this project, we had no prior information about tool use evolution that we wished to use, and indeed wished to make no prior assumptions about these dynamics. We therefore use 'uninformative' priors that reflect an absence of prior assumptions. Specifically, our priors were uniform over all

transition weights $m_{ij} \sim U(-10, 10) \, \forall \, i, j$; the prior ordering plot is thus a set of uniform distributions over time, and the prior hypercube plot is simply a hypercube with all outward edges from a given node having the same weight. HyperTraPS uses a set of simulated biased random walkers on a proposed transition matrix to characterise the probability of observed transitions; the amount of bias required to observe the required transition is recorded through each walker's progress, then this set of samples are used to compute a likelihood estimate. Here, we used $N_h = 200$ simulated walkers for each observed transition, allowing good convergence of the likelihood estimates (Johnston and Williams, 2016; Greenbury et al., 2019) (Fig. S8). MCMC chains were constructed using a Normal perturbation kernel of mean 0 and standard deviation 0.25 over each of the L^2 parameters θ (hence moving linearly in a logarithmic space of transition rates). Chains consisted of 10^6 iterations, of which 10^5 were discarded as burn-in (Johnston and Williams, 2016); we used the Gelman-Rubin diagnostic (Gelman et al., 1992) with three independent chains to test that MCMC chains were mixing well (Fig. S8).

Posterior analysis

Posterior plots were produced by simulating 10^3 random walkers on each posterior sample of hypercube edge weights. Reduced hypercube state space plots were produced by sampling the 300 most observed pathways from these simulations and recording each transition in this set of pathways. A spring-electrical force layout was used to embed the graph in two dimensions, with artificial forces separating the 'start' and 'end' states a and b. Posterior predictions for unobserved traits were obtained, as in the text, by recording every state in these simulations where *presence* markers matched those in a given observation, and recording the state of each other trait (Williams et al., 2013). Posterior predictions for future steps were obtained by recording the next step taken after a given state was reached, recorded over all simulated walkers.

References

Gelman, A., Rubin, D. B., et al. (1992). Inference from iterative simulation using multiple sequences. *Statistical Science*, 7(4):457–472.

Greenbury, S. F., Barahona, M., and Johnston, I. G. (2019). HyperTraPS: Inferring Probabilistic Patterns of Trait Acquisition in Evolutionary and Disease Progression Pathways. *Cell Systems*, 9:1–13.

Johnston, I. G. and Williams, B. P. (2016). Evolutionary inference across eukaryotes identifies specific pressures favoring mitochondrial gene retention. *Cell Systems*, 2(2):101–111.

Shumaker, R. W., Walkup, K. R., and Beck, B. B. (2011). *Animal tool behavior: the use and manufacture of tools by animals*. JHU Press.

Williams, B. P., Johnston, I. G., Covshoff, S., and Hibberd, J. M. (2013). Phenotypic landscape inference reveals multiple evolutionary paths to C_4 photosynthesis. *Elife*, 2:e00961.
